# Co-creating Inclusive and Non-ableist Public Health Policies With Persons With Disabilities: Comment on "How Did Governments Address the Needs of People With Disabilities During the COVID-19 Pandemic? An Analysis of 14 Countries’ Policies Based on the UN Convention on the Rights of Persons With Disabilities"

**DOI:** 10.34172/ijhpm.8694

**Published:** 2025-01-15

**Authors:** Janet Njelesani

**Affiliations:** Department of Occupational Therapy, New York University, New York City, NY, USA.

**Keywords:** Disability, Policy, Participatory Research, Human Rights

## Abstract

In their study, Shikako et al analyzed how national policies during the COVID-19 pandemic either supported or neglected the rights of persons with disabilities, aiming to inform the development of inclusive policies that align with the United Nations Convention on the Rights of Persons with Disabilities (UNCRPD). They concluded that the differences in policies across countries during the COVID-19 pandemic indicate a need for greater alignment and standardization of policy responses for individuals with disabilities. While the study revealed disparities across countries and underscored the importance of disability-inclusive policy, this commentary provides actionable insights to guide governments in creating equitable policies that uphold the rights of persons with disabilities during crises and beyond. Specific recommendations in accordance with the UNCRPD include the establishment of permanent consultative committees, adopting a shared understanding of disability, addressing intersectionality and structural barriers, and utilizing non-ableist participation methods so that a diverse range of perspectives are incorporated and lived experiences shape the policies that impact them.

## Introduction

 The study by Shikako et al critically examines how national policies during the COVID-19 pandemic either supported or neglected the rights of persons with disabilities.^1^ Utilizing the United Nations Convention on the Rights of Persons with Disabilities (UNCRPD) as a framework, the research assessed the alignment of national COVID-19 policies with UNCRPD commitments, identifying gaps, and recommending improvements for future policy development across 14 countries. The study analyzed 764 national COVID-19 policy documents, offering valuable cross-country comparisons of government responses to the needs of persons with disabilities. The findings revealed significant variability in policies, with six countries producing disability-specific policies. High-income countries generally had more comprehensive policies than low-income countries, reflecting differences in resources and prioritization. Most policies primarily addressed public health measures, often overlooking the broader social and economic supports required by persons with disabilities. The most frequently referenced UNCRPD Articles were Article 11 (risk and humanitarian emergencies), Article 23 (home and family), Article 24 (education), and Article 19 (community living), aligning with previous studies on the realization of disability rights in low- and middle-income countries.^2,3^

## Gaps in Policy and Participation

 Only six out of the fourteen countries produced disability-specific documents, indicating a lack of a standardized global approach to disability inclusion in COVID-19 policy-making. This also suggests that persons with disabilities and their representative organizations were likely not included in the policy decision-making process within most countries. The UNCRPD explicitly mandates the inclusion of persons with disabilities in decision-making processes that affect their lives. Articles 4 and 29 of the UNCRPD emphasize the importance of active participation of persons with disabilities in public affairs, including policy-making. This is not only a legal requirement but also a moral imperative, as it respects the autonomy and agency of persons with disabilities, ensuring that policies are reflective of their lived expertise.

 The participation of persons with disabilities in research and policy development is crucial for creating inclusive, non-ableist, and effective policies. Without the input of persons with disabilities, policy development can become ableist and exclusionary.^4^ This exclusion results in a lack of diversity in captured experiences, leading to potentially disempowering, dehumanizing, ineffective, and harmful policies. For example, as noted in the paper, during the COVID-19 pandemic, policies in many countries that did not consider the accessibility of information and healthcare services left many persons with disabilities without vital rehabilitation, mental health support, and education services, leading to significant hardships.

## Recommendations for Fostering Inclusive and Non-ableist Policy Development

 While the exclusion of persons with disabilities in decision-making processes was highlighted in the paper as noted above, with only two countries (Canada and France) having established consultative committees, the paper does not offer significant recommendations for fostering inclusive and non-ableist policy development in accordance with the UNCRPD. To address this gap of the paper, I suggest actions that must be taken up to support the development of policies that are inclusive of persons with disabilities during public health emergencies and beyond. The following suggested recommendations are drawn from my work and other scholars that have long advocated for the greater inclusion of persons with disabilities in evidence production and policy development.^4-6^

###  Collaboration With Disability Advocates and Scholars 

 Decision-makers need to collaborate with disability advocates, activists, allies, and scholars in the field of disability studies. Disability studies scholars have developed advanced methodological and theoretical insights for policy participation, demonstrating that persons with disabilities can provide unique perspectives on their rights.^7^ Participatory research and participatory action research are key approaches in this field, addressing power imbalances by prioritizing lived expertise over top-down policy development.^8^ These methodologies focus on the perspectives of those directly affected and are crucial for understanding context, revealing invisible aspects at multiple policy levels, and enhancing the value of research for both policy-makers and communities.^9^ Qualitative research, as a method used within participatory research, in particular, can explore the intersection of local lived experiences, environments, practices, and the broader political, economic, and social realities of disability. This co-generated evidence can challenge ableist attitudes and inform future policy changes, ensuring that policies are more inclusive and effective.

###  Establishment of Permanent Consultative Committees

 The establishment of consultative committees, as seen in the policies of France and Canada, is a positive step towards inclusive policy development. These committees provide a structured platform for persons with disabilities to contribute to policy-making, ensuring their perspectives are heard and their needs addressed. However, the limited presence of such committees in other countries studied indicates a need for broader institutionalization of these practices globally. I therefore suggest that Governments should value the contribution of individuals with lived experience by institutionalizing the participation of persons with disabilities in policy-making processes through the establishment of permanent consultative bodies. These groups, which may take the form of advisory bodies or representatives from disability organizations, collaborate with decision-makers to co-create knowledge.^10^ To address power hierarchies and develop policies responsive to the diverse needs of persons with disabilities, these bodies should include individuals from diverse backgrounds to ensure comprehensive representation. As an example, in the United States, the President’s Committee for People with Intellectual Disabilities and the National Council on Disability provide valuable models. Although the U.S. was not included in the study sample, examining its efforts offers insights of the potential and challenges of consultative committees in integrating the voices of people with disabilities into policy-making. The President’s Committee for People with Intellectual Disabilities serves as a federal advisory body to the President and the Secretary of Health and Human Services, focusing on policies and programs that impact individuals with intellectual disabilities. This committee ensures that the perspectives and needs of this population are considered at the highest levels of governance. Similarly, the National Council on Disability advises the President, Congress, and executive agencies on policies designed to uphold and advance the goals of the Americans with Disabilities Act.

###  Adopting a Unified Framework for Understanding Disability

 While the creation of consultative committees is vital, a unified framework for defining, understanding, and measuring disability across diverse fields, expertise, communities, and policy contexts is also needed. Historically, because of the lack of consistent definitions and indicators of disability, combined with differences in methodologies used to gather data there have been major challenges in producing reliable and comparable disability statistics to inform policy.^11,12^ Without a shared understanding of disability, efforts to ensure that these committees are representative risk not aligning with the lived experiences and rights of people with disabilities. By adopting a shared understanding, such as the International Classification of Functioning, Disability and Health biopsychosocial approach to conceptualizing disability,^13^ policy-makers and stakeholders can address disability more holistically and inclusively as there would be greater coherence among policies and programs across sectors.

###  Acknowledging Intersectionality in Disability Policy

 Establishing diverse consultative bodies acknowledges that disability is multifaceted and aligns with the authors’ findings that many policies have overlooked the intersectionality of disability with other identities and socio-economic factors such as gender, ethnicity, and economic status. Such policies often neglect the specific needs of various subgroups within the disability community. To address intersecting factors and tailor policy responses that fully address the range of rights, individuals with disabilities from diverse backgrounds must be included in the policy-making processes to develop more nuanced and effective policies. Currently, the participation of people with disabilities in policy development often prioritizes recruitment based on medicalized conceptions of competence. As a result, many individuals within the disability community, such as those who use augmentative communication or have intellectual disabilities, are excluded from research and policy development.^14^ This exclusion means that many perspectives remain unheard, and their lived experiences do not influence the policies that impact them. As suggested above, the International Classification of Functioning, Disability and Health framework could serve as an appropriate foundational framework as it recognizes that not all persons with disabilities are equally restricted in their participation. For example, girls with disabilities experience the combined disadvantages associated with gender as well as disability and may be less likely to go to school than non-disabled girls.

###  Non-ableist Methods for Inclusive Policy Development Participation

 Inclusive participation of persons with disabilities necessitates non-ableist methods. Significant barriers to meaningful participation include ableist cultures that foster discrimination and low expectations, which often reduce contributions to mere narratives of experience rather than valuing expertise. Other barriers include issues related to access and accessibility, and the need for structural support, opportunities, appropriate information and resources, and skill development.^4^ Meltzer et al have created a practical framework to aid policy-makers in incorporating lived experiences.^15^ This framework is based on inclusive, participatory, and action research, and it addresses access and accessibility challenges. The framework includes three key principles for the inclusion of persons with disabilities; invite meaningful and flexible participation, make information accessible, and amplify voices to ensure policies can respond to what people actually say they need, not what policy-makers may perceive they need. Ensuring accessibility is crucial to maintaining inclusivity across a wide spectrum of disabilities and needs, and to prevent a narrow view of whose perspectives are considered valuable. To meet these accessibility rights, policy-makers must develop skills and receive training on disability rights principles. All aspects of policy-making and implementation must be accessible to persons with disabilities, including public consultations, information dissemination, and service delivery. This involves providing materials in accessible formats, such as braille, sign language interpretation, and easy-to-read formats.

## Future Research Directions

 Future studies would benefit from placing the examined pandemic responses within the broader historical and social context of disability rights and policy development. It is essential to consider how historical attitudes and policies, both within each country and globally, have shaped the lived experiences of persons with disabilities. Comparing pandemic responses to past public health crises and examining the evolution of disability policies would provide a richer, more contextualized analysis. This approach would also enable researchers to make culturally attuned recommendations for the countries included in the study.

## Conclusion

 In conclusion, Shikako and colleagues’ study offers a thorough analysis of the alignment of COVID-19 policies with the UNCRPD across 14 countries. The findings highlight the need for a more inclusive, comprehensive, and standardized approach to policy-making that fully incorporates the rights of persons with disabilities, ensuring their protection and well-being during crises. Involving persons with disabilities in developing national policies is not merely a matter of legal compliance but an essential step toward achieving inclusivity and equity. Duty bearers must acknowledge and act on this imperative to create a more just and supportive society for all individuals. This commentary provides valuable insights for policy-makers on how to develop inclusive, non-ableist policies. By institutionalizing the participation of persons with disabilities, adopting an intersectional approach, and ensuring accessibility, governments can better protect and promote the rights of persons with disabilities, particularly during crises.

## Ethical issues

 Not applicable.

## Conflicts of interest

 Author declares that she has no conflicts of interest.
